# A Systems Approach Uncovers Restrictions for Signal Interactions Regulating Genome-wide Responses to Nutritional Cues in Arabidopsis

**DOI:** 10.1371/journal.pcbi.1000326

**Published:** 2009-03-20

**Authors:** Gabriel Krouk, Daniel Tranchina, Laurence Lejay, Alexis A. Cruikshank, Dennis Shasha, Gloria M. Coruzzi, Rodrigo A. Gutiérrez

**Affiliations:** 1 Center for Genomics & Systems Biology, New York University, Department of Biology, New York, New York, United States of America; 2 Departamento de Genética Molecular y Microbiología, Pontificia Universidad Católica de Chile, Alameda, Santiago, Chile; 3 Courant Institute of Mathematical Sciences, New York University, New York, New York, United States of America; 4 Institut de Biologie Intégrative des Plantes, UMR 5004, Biochimie et Physiologie Moléculaire des Plantes, Agro-M/CNRS/INRA/SupAgro/UM2, Montpellier, France; UT Southwestern Medical Center, United States of America

## Abstract

As sessile organisms, plants must cope with multiple and combined variations of signals in their environment. However, very few reports have studied the genome-wide effects of systematic signal combinations on gene expression. Here, we evaluate a high level of signal integration, by modeling genome-wide expression patterns under a factorial combination of carbon (C), light (L), and nitrogen (N) as binary factors in two organs (O), roots and leaves. Signal management is different between C, N, and L and in shoots and roots. For example, L is the major factor controlling gene expression in leaves. However, in roots there is no obvious prominent signal, and signal interaction is stronger. The major signal interaction events detected genome wide in *Arabidopsis* roots are deciphered and summarized in a comprehensive conceptual model. Surprisingly, global analysis of gene expression in response to C, N, L, and O revealed that the number of genes controlled by a signal is proportional to the magnitude of the gene expression changes elicited by the signal. These results uncovered a strong constraining structure in plant cell signaling pathways, which prompted us to propose the existence of a “code” of signal integration.

## Author Summary

Light (L), nitrogen (N), and carbon (C) are well known to be strong signals regulating gene expression in plants. But, so far, few reports have described their interactions on a genome scale. Here, we report the transcriptome response of the factorial combination of these three signals in leaves and roots of *Arabidopsis*, corresponding to all possible combinations or 16 different treatment conditions. To mine this complete transcriptome data set, gene expression was modelled as a function of the C, N, L, and O (organ) signals. This computational approach revealed that multiple signals coordinate gene expression precisely and according to a constrained plan, which we call the “code of signal interaction.” Our studies indicated that signal integration occurs differently in different organs. We identified new modes of signal interaction that imply existence of new signaling pathways coordinating gene expression on a genomic scale.

## Introduction

Living organisms need to integrate both internal and external signal information in order to program the appropriate responses for survival. Signaling pathways that respond to single nutrient or hormonal signals are on the way to being resolved [1],[2],[3],[4],[5],[6],[7],[8]. However, little is known about how multiple signals are integrated on a genome-wide scale to change gene expression, make physiological adjustments and/or direct new programs of development. In plants, some early clues to these molecular mechanisms come from the study of hormonal crosstalk [9],[10]. The prevalence of multiple hormone-resistant mutants suggests that such crosstalk is very frequent [11]. In plant nutrition, it has been clearly established that proteins involved in glucose sensing (HXK1), nitrate transport (NRT1.1, NRT2.1) and light signaling (HY5) are involved in the crosstalk with auxin/cytokinin [12], auxin [13],[14],[15] and abscisic acid signaling [16], respectively. This crosstalk is proposed to allow regulation of growth to be tuned to nutrient or light availability. However, very few of the molecular elements generating crosstalk between nutritional signaling pathways are known. For instance, Carbon (C), Light (L) and Nitrogen (N) signals are well known to be finely coordinated to ensure the appropriate Carbon/Nitrogen ratio (C/N) needed for amino acid synthesis under a specific light regime. In particular, N transport and assimilation genes are known to be under the control of L/C/N signals [17]. For genes encoding transporters, this C/L control can involve different C-related signaling pathways [18]. It has also been demonstrated that photosynthetic genes are under regulation by N and C [12],[19]. Previous genome-wide studies have shown that C, N and C/N control major cellular functions such as energy, metabolism, C-metabolism, and fundamental processes such as ribosome biogenesis [20],[21],[22]. Together, the evidence indicates a strong coordination between the C/N/L signals. However, the underlying mechanism(s) and models of signal integration involved in this crosstalk have yet to be proposed.

Recently, a bioinformatics approach was undertaken to characterize the crosstalk between seven different hormones [23]. By analyzing lists of hormone-responsive genes, the authors concluded that a very low level of interaction between hormone signaling pathways exists because of the small overlap among these lists. However, they do predict that the biosynthesis of each hormone is susceptible to control by others, which has been recently proven for ethylene-controlled auxin synthesis [24],[25].

In our study, we integrate experimental and bioinformatics analysis to evaluate interactions of nutrient and light signals, using gene expression as a reporter of signal effects. For this, we analyzed the *Arabidopsis* transcriptome (using Affymetrix ATH1 GeneChips) under a complete factorial combination of Carbon (C), Nitrogen (N) and Light (L) on two different Organs (O), roots and shoots. The response of each gene was modeled as a function of each factor (C, N, L, O) and all possible interactions using analysis of variance (ANOVA). Thus, if a gene is controlled for instance by N and C, it constitutes a marker of convergence for signals from these two factors. By considering the whole set of regulated genes (a third of the genome), this logic allowed us to follow signal interaction on a genome-wide scale. This quantitative vision of factor interactions allowed us: i) to discover an unexpectedly strong level of signal integration that we consider to be a ‘code’ of gene expression control; ii) to decipher major relationships between factors (C, N, L, O) on a genomic scale; and iii) to uncover a characteristic of signal propagation, linking the number of genes controlled by a signal to the magnitude of its control on individual gene expression.

## Results

### Genome-wide analysis of gene expression responses to Carbon (C), Nitrogen (N), Light (L) and Organ (O)

**Figure 1:**
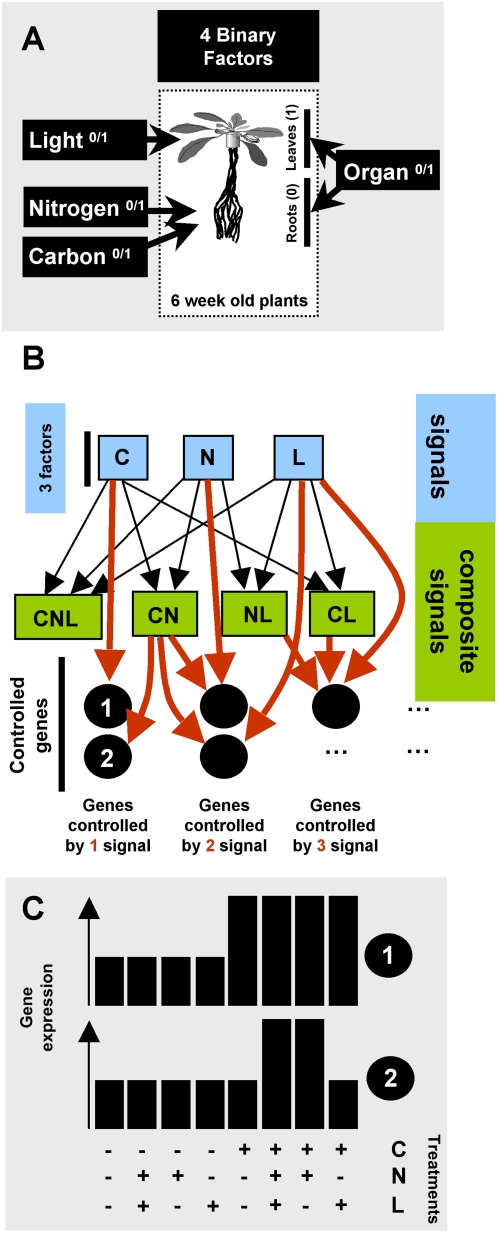
Scheme of experimental design and working model of gene control by multiple signals at the organ-specific level. A) 6-week-old plants were treated for 8 h with all combinations of three (C, L, N) binary (0/1) factors. Leaves and roots were analyzed separately for a total of 16 experimental conditions. Treatments were as follows: N, 5 mM NO_3_^−^; C, 30 mM sucrose; L, 60 µmol.m^−2^.s^−1^. RNAs were extracted from roots and shoots separately and hybridized to ATH1 Affymetrix chips. Microarray data analysis was performed as described in Experimental Procedures. B) Scheme presenting the concept used to decipher signal interactions in the control of gene expression. We propose that perceived signals can be produced from a factor (C, N, L represented as blue squares) or combination of factors (green squares). These combination of factors build what we name “composite signals”. These signals or composite signals can then affect the expression of a particular gene. The expression of a gene (e.g. black circles labeled 1 and 2) can be affected by (red arrow) one signal (e.g., C alone for number 1) or a composite signal (e.g., C and N for number 2). C) Idealized gene expression patterns produced by the signal effects shown in (B) for the genes 1 and 2.

We analyzed global gene expression patterns in all possible combinations of C, L and N as binary factors (presence or absence) on two different organs (leaves and roots). Plants were grown hydroponically in L/D cycles (8/16 h) for six weeks, with 1 mM nitrate as the N source and without exogenous C. They were then treated for 8 h with combinations of 30 mM sucrose, 5 mM nitrate either in the light (60 µmol.m^−2^.s^−1^) or in darkness. Those conditions were chosen according to our previous study [20] in which we showed that neither gene expression nor signal interaction could be correlated to the quantity of nitrate or sucrose provided. We thus chose to use the lowest concentrations of the nutrients previously tested to minimize osmotic effects. Roots and leaves were harvested separately and used for total RNA isolation. This strategy corresponds to 16 different experimental conditions, including organ as a factor (Figure 1A). RNA samples were used to hybridize the Arabidopsis ATH1 genome array from Affymetrix to evaluate global gene expression. All experiments were performed in duplicates. All hybridizations were normalized using the MAS*v*5.0 package and analyzed with custom-made R functions. To evaluate the effect of the experimental treatments on gene expression, we used ANOVA on the expression of each gene represented on the microarray. We used two different models for ANOVA analysis. The first model considers the organ as a factor, such that the expression Y_i_ of a gene_i_ is given by: Y_i_ = α_0_+α_1_C+α_2_L+α_3_N+α_4_O+α_5_CL+α_6_CN+α_7_CO+α_8_LN+α_9_NO+α_10_LO+α_11_CNL+α_12_LNO+α_13_CNO+α_14_CLO+α_15_CLNO+Z. In this model, α_0_ represents the expression under a “control” condition (without C, without N, without L, in roots), *Z* represents the noise, and α_1_ to α_15_ represent the coefficients quantifying the effect of each factor (C, N, L, O) or combination of factors. For example, the coefficient of CNL represents the effect of C, N and L in combination, over and above the main effects of C, N, L and O, and all two-way interactions among these factors. The second model is just a simplified version of the first model in which gene expression in the root and leave datasets were analyzed separately: Y_i_ = α_0_+α_1_C+α_2_L+α_3_N+α_4_CL+α_5_CN+α_6_LN+α_7_CNL+Z. These two modeling approaches were used because they highlight three different aspects of the data (1, whole data set; 2, leaves only; 3, roots only). Indeed, we found that the O effect is a predominant factor that controls gene expression (see below) and that its dramatic effect on gene expression can mask the weaker effects of other factors. On the other hand, the analysis of the whole dataset provides insight into how the O factor is integrated and how it influences the other factors. The results of the modeling are provided as Table S1 for the whole dataset, Table S2 for leaves and Table S3 for roots. These tables summarize the significant coefficients (i.e. magnitude of the effect) for each factor or combination of factors in the model for each gene and constitute the basis for further analyses. Note here that in the following analyses, we considered that each factor (C, N, L, O) can be the *signal* triggering gene regulation on its own. Furthermore, combinations of factors (such as for instance NL), named *composite signals*, can be the necessary condition for a gene to be regulated (illustrated Figure 1B and 1C). This terminology (signal *vs* composite signal) is used throughout the manuscript and discussed below for its physiological consequences. From the modeling using the entire dataset, 8,036 genes (35% of the genome) were found to be significantly controlled by at least one factor or combination of the four factors. We found 3,279 (14.3%) and 1,002 (4.4%) genes that were regulated by at least one factor (C, N, L) or combination of factors in leaves and roots respectively.

### A ‘code’ of signal interaction?

**Figure 2:**
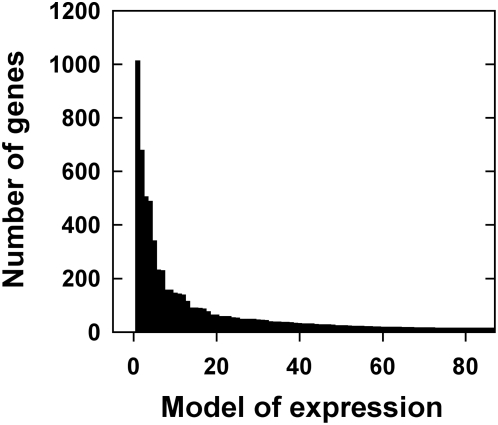
A small number of models explain most gene expression patterns in response to 16 different experimental conditions. The gene expression patterns obtained from the 16 different experimental conditions were modeled as a function of the four experimental factors and their interactions using a rigorous statistical procedure (see Materials and Methods). Genes with the same model of expression were grouped. The graph shows the number of genes (Y-axis) explained by the different models of gene expression (X-axis).

To understand the global patterns of response to the experimental factors, we simplified the matrices with the gene expression models described in the previous section using a binary code. We replaced model coefficients that were negative, not significant or positive with a −1, 0 or 1, respectively. Thus, genes harbouring similar expression patterns (successions of 0, 1 or −1) could be grouped in the same model of regulation (independent of the magnitude of the effect). Considering the whole data set, a gene can be either induced, repressed or not affected by the 15 terms (C, L, N, O, CL, CN, CO, LN, NO, LO, CNL, LNO, CNO, CLO, CLNO) derived from the combinations of the 4 factors and their 1^st^, 2^nd^, and/or 3^rd^ order interactions. Thus, a gene can respond in any one of 3^15^ = 14,348,907 possible ways. Our global analysis led to the surprising result that a very large number of genes are controlled by a very small number of regulation models (Figure 2, Table 1 as truncated version; Table S4 as full version). For instance, we found that 6,422 out of the 8,036 regulated genes (79.9%) are explained by only 87 of the 3^15^ possible models of gene regulation. This result indicates that there is a major constraining structure in plant cell signaling pathways. We thus hypothesize the existence of a ‘code’ governing signal integration at the organism level, which is responsible for the observed global gene expression reprogramming in response to C, N and L in two different organs. Indeed, a code can be defined as “A systematically arranged and comprehensive collection of laws” (Oxford English Dictionary definition). In our case, if we consider the presence or the absence of the studied factors and their interactions (as an input), the gene expression (the “output”) is deterministic and driven by *a comprehensive collection of law*. We thus propose that this structure can be compared to/defined as a “code” of signal interaction controlling gene expression.

**Table 1:** Predominant model of expression at the whole data set level.

C	N	L	O	CN	CL	CO	LN	NO	LO	CNL	LNO	CNO	CLO	CNLO	#genes
0	0	0	−1	0	0	0	0	0	0	0	0	0	0	0	**1009**
0	0	0	1	0	0	0	0	0	−1	0	0	0	0	0	**676**
0	0	0	1	0	0	0	0	0	1	0	0	0	0	0	**502**
0	0	0	0	0	0	0	0	0	1	0	0	0	0	0	**485**
0	0	0	1	0	0	0	0	0	0	0	0	0	0	0	**337**
0	0	1	−1	0	0	0	0	0	0	0	0	0	0	0	**229**
0	0	−1	−1	0	0	0	0	0	0	0	0	0	0	0	**226**
−1	0	−1	−1	0	1	1	0	0	1	0	0	0	−1	0	**154**
0	0	1	0	0	0	0	0	0	−1	0	0	0	0	0	**153**
0	0	−1	−1	0	0	0	0	0	1	0	0	0	0	0	**142**
0	0	1	0	0	0	0	0	0	0	0	0	0	0	0	**139**
1	0	1	0	0	0	−1	0	0	0	0	0	0	0	0	**135**
1	0	1	0	0	0	−1	0	0	−1	0	0	0	0	0	**112**
															**…**

Expression of each gene has been modeled as a function of C, L, N and O factors and their interactions. Each gene model was recoded replacing by −1, 0 or 1, coefficients respectively for negatively, not significant or positively regulated genes. Number of genes in each class is indicated in the last column. The full version of this table is provided in Table S4.

### Deciphering the signal interaction “code”

**Figure 3:**
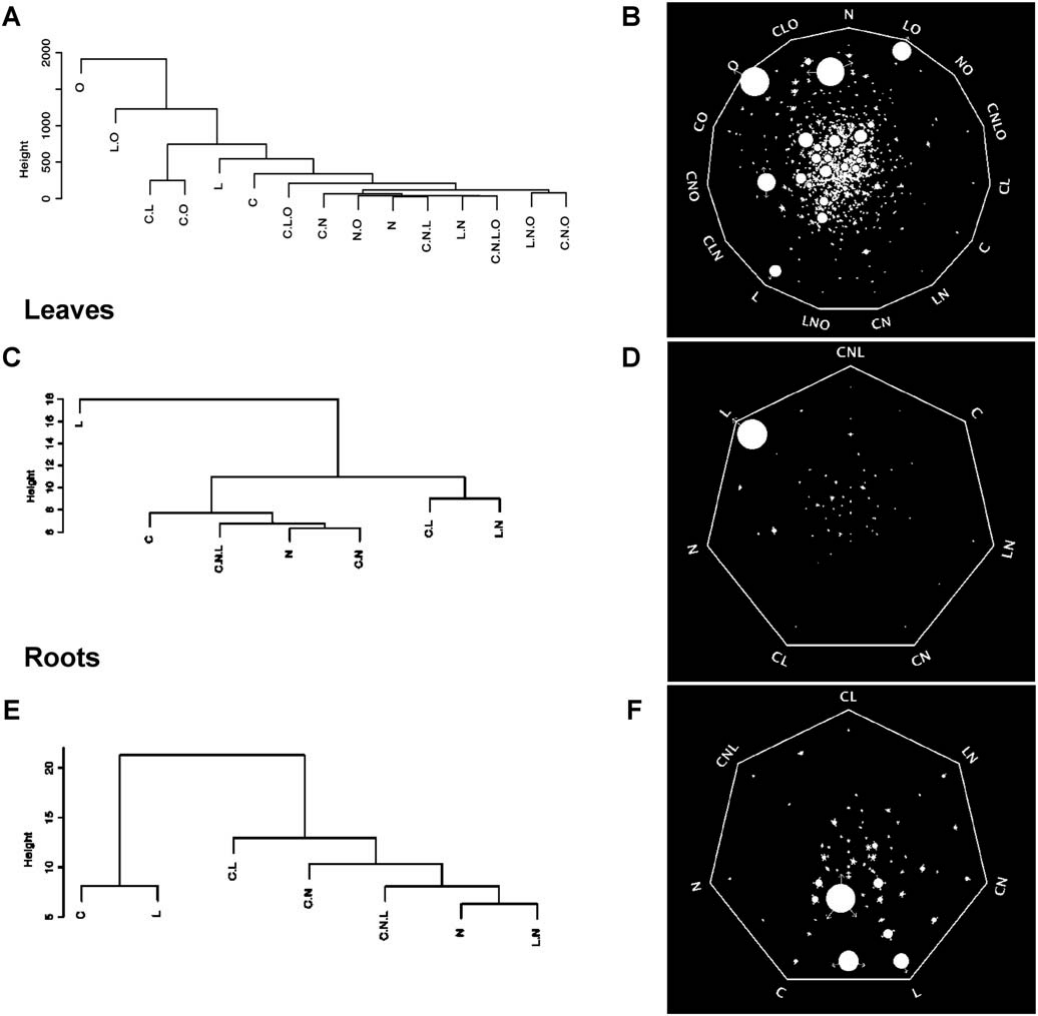
Signal strength and relationship for the control of gene expression. A, B) Analysis using the entire data set; C, D) Analysis using data from leaves; E, F) Analysis using data from roots; A–C) Dendrograms produced by average linkage hierarchical clustering analysis with euclidean distance carried out on the simplified model matrices as described in the text. B–F) Analysis of signal strength using the Sungear software. The Sungear polygon shows the signals at the vertices (anchors). The circles inside the polygon (vessels) represent the genes controlled by different signals as indicated by the arrows around the vessels. The area of each vessel (size) is proportional to the number of genes associated with that vessel. Thus, it is visually and quantitatively possible to identify the main signal at the whole dataset level as O. In leaves, L predominates, and in roots C and L are similar with regard to the number of genes affected. See details for interpretation in Materials and Methods.

To elucidate the structure that controls the regulation of gene expression by the experimental factors and their interactions, we used two approaches. The first is based on clustering across the three matrices described above (whole data, root, shoot). This method, adapted from Speed (2003), enables qualitative analysis of the co-occurrence of each term in the models of gene expression (Figure 3A,C,E)[26]. The second method uses the Sungear software [27] to quantitatively evaluate the importance of each term, as assessed by the number of genes, in the models of gene expression (Figure 3B,D,F) (Please refer to the Materials and Methods section for a detailed explanation on clustering and Sungear software use). Thus, we used average linkage hierarchical cluster analysis with euclidean distance on the simplified matrix of regulatory models (Table S4). To do so, we multiplied each column in Table 1 by the number of genes with the corresponding model (last column in Table 1) to weight each row proportionally to the number of genes. The dendrograms generated by the clustering algorithm allowed us to infer the relationship between the signals and/or the composite signals (as defined in Figure 1) in the control of gene number (Figure S1) [26]. To evaluate the signal strength as determined by the number of genes controlled by each signal we also used Sungear, which is a software tool designed for the dynamic analysis and visualization of multiple lists of genes [27],[28] (See Materials and Methods section for detailed description of the Sungear tool). In a second analysis, we used hierarchical clustering analysis on the model coefficients (Tables S1; S2; S3). In this case, we grouped signals based both on their relationship and magnitude of their effect on gene expression. The combined hierarchical clustering and Sungear analysis revealed that O is the predominant factor controlling gene expression (Figure 3A and 3B). In leaves, the main signal is L (Figure 3A–3D), while in roots the L effect manifests as an interaction with C (Figure 3E, 3F). That is, genes controlled by L in leaves do not typically respond to other signals, but in roots genes controlled by L are also largely controlled by C. This logic can be used to decipher the relationships and strengths of any of the signals or composite signals (Figure 3).

**Figure 4:**
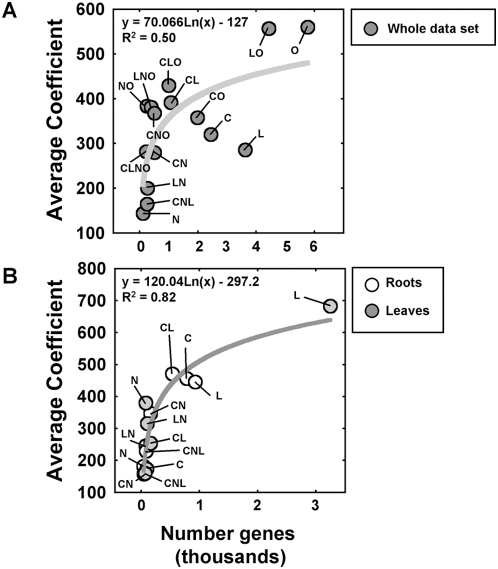
Relationship between the number of regulated genes and the magnitude of gene regulation (coefficients of the model). The graphs show the relationship between the average coefficient and the number of genes that showed the coefficient as significant in the regulation model. Circles are labeled with the corresponding signal. The coefficient of determination (R^2^) for each logarithmic regression analysis is indicated in the graphs. (A) Analysis for the complete data set. (B) Analysis for roots and leaves data sets separately.

Interestingly, the hierarchy of signals and composite signals in this analysis seems to be comparable to our first analysis based on model size (compare dendrograms in Figure 3 and Figure S1). This finding suggested that for a given signal, its strength on individual gene regulation and the number of genes in the genome that are controlled by this signal are correlated. To test this hypothesis, we plotted the absolute values of the model coefficient (an indicator of the strength of regulation) against the number of genes controlled by each individual signal or composite signal (Figure 4). We observed a logarithmic relationship between these two parameters at the whole dataset level (R^2^ = 0.50) and at the organ-specific level (R^2^ = 0.82) (Figure 4). Note here that logarithmic regression excluding the L signal in leaves is still very significant (R^2^ = 0.74). The two terms with the largest coefficient (i.e. largest effect on gene expression) and number of genes, C and L, seem to be the ones that behave most differently in the roots and leaves datasets. Treating data from root and leaves separately allowed us to reduce this constraint and improved the regression. Thus, if we sort the signals and the composite signals by their ability to control gene expression, two components can be identified. The first component encompasses weaker interactions, controlling few genes (<500 genes). In this component, the strength of the signal increases without a concomitant increase in the number of genes regulated. In the second component (>500 genes), we observe the inverse relationship. The strength of the regulation reaches a ‘plateau’ (at a value of approximately 450 in the coefficients), but there is a large increase in the number of regulated genes (Figure 4).

### The rules of signal integration

**Figure 5:**
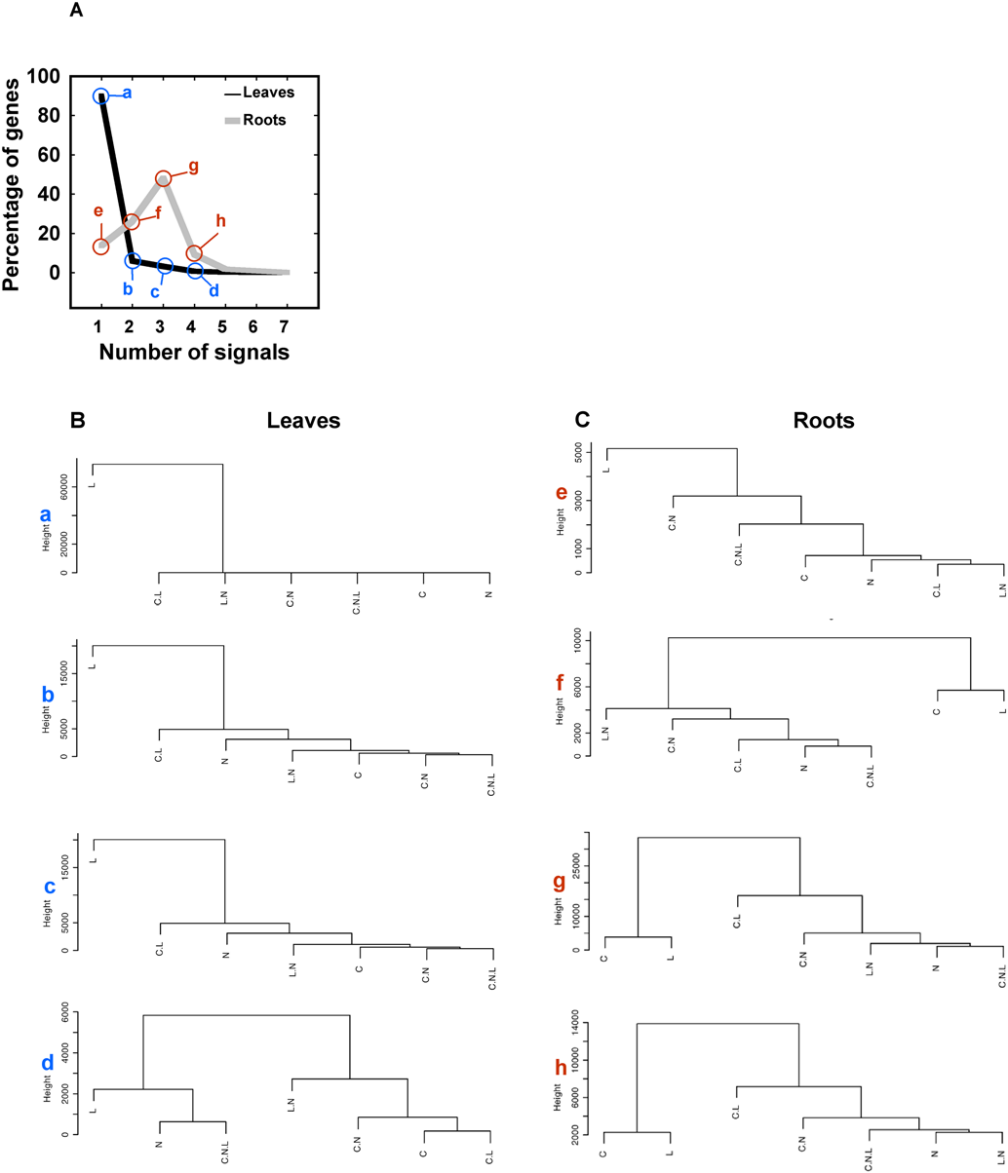
Signal integration at the organ-specific level. A) Percentage of regulated genes as a function of the number of signals. In leaves most genes are regulated by only one signal, labeled with the letter “a”. Genes belonging to the groups labeled with letters (a to e) in panel A were subjected to average linkage hierarchical clustering with euclidean distance to analyze the signal relationship across increasingly complex models of gene expression in leaves B), in roots C). Dendrograms show hierarchy of signals in the control of gene expression (a to d for leaves, e to h for roots).

To gain a better understanding of how plants respond and integrate multiple experimental factors, we analyzed the number of genes controlled by *x* number of signals or composite signals (as defined in Figure 1B, 1C). This analysis revealed that signal integration is stronger in roots than in leaves (Figure 5A). In leaves the large majority (89.8%) of genes are controlled by only one factor, whereas in roots, 86.2% of genes are controlled by two or more factors (Figure 5A). To decipher the relationships underlying the dichotomy between leaves and roots, gene lists corresponding to each group (a to h in Figure 5A) were subjected to hierarchical clustering (Figures 5B and 5C). This approach showed that in leaves 99.6% of the 89% of the genes controlled by only one signal are controlled by L (Fig 5B a). Therefore, L responses in leaves are mostly independent of the other signals. In roots, genes with simple models with one significant term also show a dominance of L (78% are induced by light only; Figure 5C e). However, as the models become more complex (Figure 5B e to h), L and C appear related (compare Figures 5C e to h). Furthermore, the effect of N is mainly observed as an interaction with C and L, indicating that the effect of N is largely dependent on the context of the other signals. This result is consistent with previous studies that indicate a large component of the N-response was dependent on the particular conditions used in the experiment [26].

**Figure 6:**
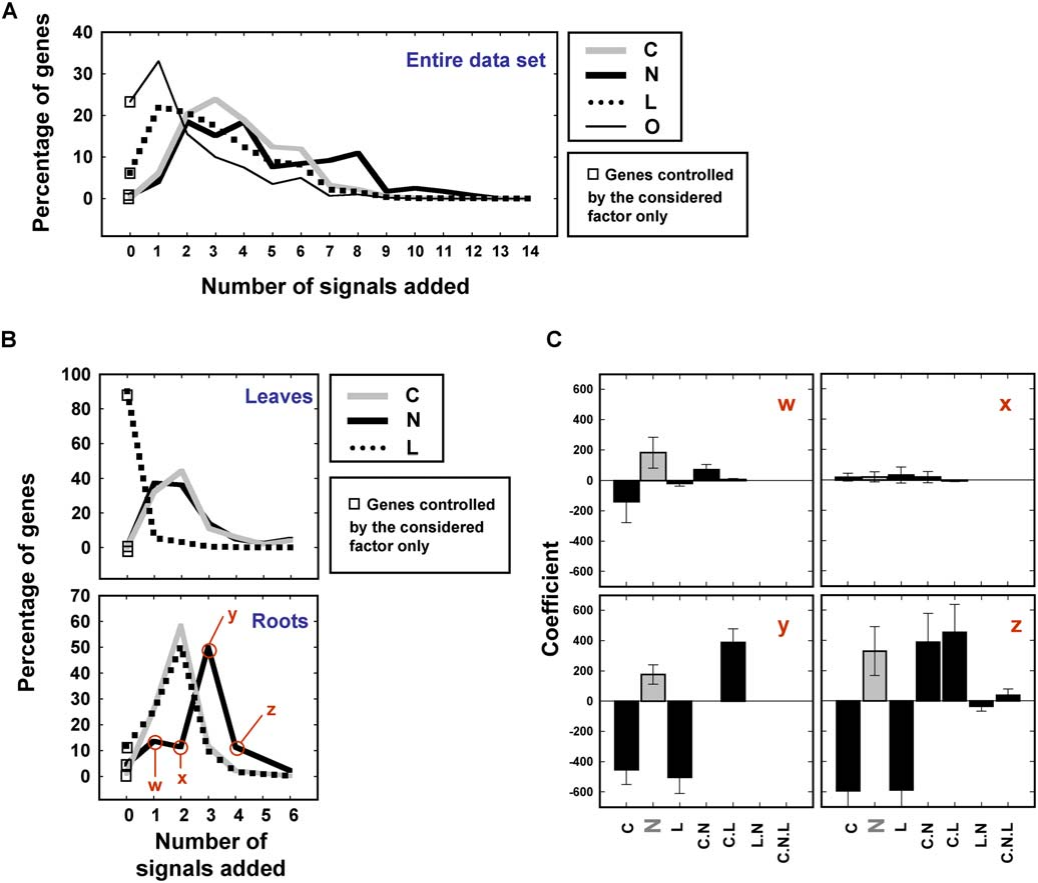
Signal integration of C, L, N and O factors: case of study for nitrogen. Effect of added signal on the percentage of controlled genes considering each factor at A) the whole dataset level or B) the organ specific level. C) Centroid-plots of model coefficients for the gene lists (w,x,y,z) considered in (B). Note that in C-x, the N effect is significant (by definition), but no trend between genes gathered in the list can be visualized. Some of the genes are positively controlled by N, others are negatively controlled.

To further characterize signal cross-talk in our conditions, we analyzed the number of genes controlled by a given signal (C, N, L or O) and the effect of adding *x* other signals or composite signals (Figure 6). This approach provides information about how signals superimpose to control gene expression at the whole plant (Figure 6A) and organ-specific (Figure 6B) levels. In leaves, most genes are regulated by L alone (Figure 6B). In contrast, genes that respond to N or C are also regulated by one or two additional signals or composite signals (Figure 6B). No gene was found to be controlled by N or C alone, indicating that N and C are mainly sensed as composite signals rather than as single signals in leaves. In roots, most genes regulated by C, N or L are under the control of at least two other signals (Figure 6B).

To conclude the analysis of signal cross-talk, we evaluated the patterns of signal interactions. For example, to identify the signal(s) that interact with N in roots we analyzed the coefficients of the ANOVA models (indicating the direction and strength of the regulation) that included N (Figure 6C). We found that ANOVA models that included N and tree other signals were similar (Figure 6B and 6C, y and z data points and panels respectively). These N-controlled genes are negatively controlled by C and L signals and positively controlled by the CL composite signal in roots (100% of the 22 genes in this gene list follow this same pattern). This is not the case for simpler models such as N controlled by one or two additional signals or composite signals (Figure 6b and 6C, w and x data points and panels respectively). A summary of all patterns found is provided in the following section.

### A model of signal integration in roots

**Figure 7:**
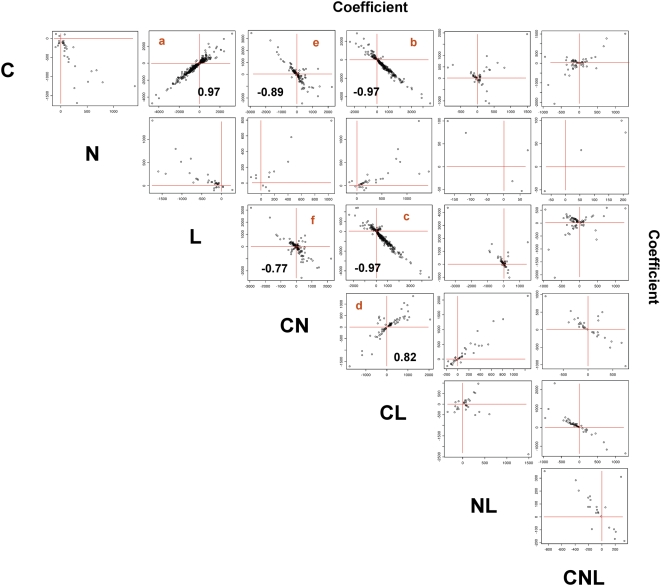
Signal integration in roots. Genes controlled by at least each pair of considered signals were identified and then plotted based on their gene expression ANOVA coefficients. Significant Pearson correlation coefficients are presented in corresponding panels (a–e).

**Figure 8:**
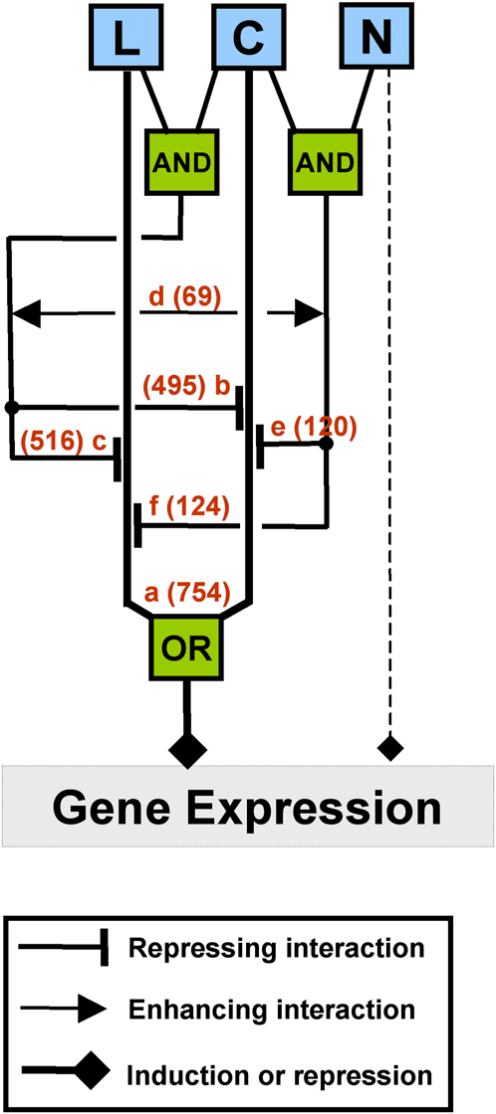
Conceptual model of signal interactions in Arabidopsis roots. The strong relationships discovered (a–e) in Figure 7 were summarized by a conceptual model. Number of genes involved are provided on the top of arrows. More details are provided in Materials and Methods section.

To identify general patterns of signal integration, we analyzed the relationship between each pair of signals or composite signals. The ANOVA coefficients for each pair of signals or composite signals in a model were plotted against one another (Figure 7). For example, the second panel in the first row of Figure 7 (labelled a) shows the values of the coefficients for models that contain both C and L signals plotted against each other. This analysis indicates a high correspondence for the effect of C and L on gene expression in roots. In this case, the influence of L was positively correlated with the influence of C, consistent with the hypothesis that L is mainly sensed as sugars in roots (Figure 7 a). Similar analysis reveals that C signals are inversely correlated with CL and CN signals. This indicates that the C effect is reduced in the presence of L or N (Figure 7 e-b). The effect of L is reduced by the presence of C or N (Figure 7 f-c). The significant relationships between signals (more than 50 genes with Pearson coefficient>0.80) were used to draw regulatory relationships that were summarized in a model of signal integration (Figure 8). In Figure 8, we use logic gates to represent the effect of each signal on gene expression. For instance, the presence of C *OR* L has the same effect on the expression of 754 genes that are regulated by these signals. Similarly, we used AND gates to represent that two signals are required for an effect on gene expression. For example, the presence of C *AND* N is needed to repress the effect of the L signal on gene expression. This conceptual model of signal interaction in Arabidopsis roots is discussed further for its predicted physiological consequences.

## Discussion

### Four factor factorial design: The key to ‘code’ discovery

For the past decade, transcriptome studies have been used to understand molecular events involved in responses to biotic, abiotic or hormonal treatments or developmental series (for an overview see https://www.genevestigator.ethz.ch/ or http://bbc.botany.utoronto.ca/efp/cgi-bin/efpWeb.cgi). Nevertheless, only three reports have systematically addressed the interaction between experimental factors genome-wide (C *vs* N, C *vs* L) [20],[22],[29]. These approaches revealed gene networks involved in plant adaptation to a fluctuating N, C and L environment. Here, increasing the number of factors to four (C, N, L, O) allowed us to reach a new level of complexity. When analyzing single factors, there are 3^1^ different models possible (induced, repressed or not regulated). This same logic (depicted Figure 1B) applies to two factors (3^3^ = 27 different models), three factors (3^7^ = 2,187), four factors (3^15^ = 14,348,907) and so on. But it is only by performing the experiments with four factors that we uncovered the tremendous constraint in signaling pathways in Arabidopsis. In the systematic analysis of this dataset, we found that the distribution of gene expression patterns fell within very few models of expression and revealed a strong coordination between signals. The probability of finding the observed models by chance is negligible (<10^−323^). This result supports the idea of a ‘code of signal interaction’. It is clear that our modeling approach can explain only part of the gene expression variability. However, our results suggest that plant cell signaling pathways are constrained such that the possible outputs in response to simultaneous change in multiple external factors are restricted to a very small portion of the total possibilities. Since our model, i) might miss non-linear relationships, ii) is built on data obtained from multi-cellular organs (roots and shoots), we hypothesize that the structure in plant cell signaling pathways is even more restrictive than what proposed here. For example, it could be of great interest to reproduce this analysis at the cell-specific level to unmask regulation hidden at an organ level. For a simple NO_3_^−^ treatment, cell specific analyses were successful in revealing regulation obscured from whole organ analysis [30].

### A link between the strength and the number of controlled genes by a signal

Our current analysis uncovered a relationship between the strength of signals or composite signals (absolute value of model coefficient) and the number of genes controlled by these signals (Figure 4). A recurrent logarithmic law in biology is known to link the perceived sensation/response of biological systems to true stimulus intensity. The Weber–Fechner equation [31] can be applied to many different biological systems: from human odor perception [32] and time perception [33] to prefrontal cortex neuronal activity of monkeys under visual stimulation [34] or cockroach neuron response to light intensity [35]. It is thus tempting to hypothesize that the plant transcriptome response might be under the same kind of mechanistic stimulus/perception relationship. However, our study does not directly link the strength of the applied signal, but instead two components of the sensed signals (1, number of regulated genes and 2, gene regulation magnitude). Further investigation is warranted to (i) validate this link between gene response and applied signal intensity in *Arabidopsis* and (ii) demonstrate that this strong logarithmic relationship can be found in the transcriptomes of other living organisms.

### Working model validation and finding of Boolean-like signal integration

In the proposed models to explain gene expression in response to multiple experimental factors (Figure 1), we hypothesised that plants sense combinations of signals (Figure 1B, 1C). This assumption is supported by experimental data. For instance, it as been demonstrated that *NRT2.1*/*NRT3.1* repression (coding a major component of the high affinity NO_3_^−^ transport system) is effective only when both high NO_3_^−^ *AND* high NH_4_^+^ are present in the medium [7]. Our present study also supports this point of view. Indeed, the ANOVA model that we used has uncovered genes that behave as proposed in Figure 1C. For instance, modeling of leaf data detected three genes that were controlled as a single independent composite signal by the presence of CL, two by CN (as defined in Figure 1 gene #2), or four by LN. In roots, two genes were found to be controlled by CL, nine by CN, and six by LN as a single and independent composite signal (Figure S2). This *post hoc* analysis provides support for the modeling approach and suggests that plants can sense combinations of factors as single signals. From another standpoint, this analysis suggests that genes are under the control of AND-like-logic-gates, as we previously showed for C/L and for NH_4_/NO_3_ [7],[36]. Our present study suggests that this kind of boolean-like-regulation can affect genome-wide expression in plants (Figure 7 and Figure 8). Moreover, it is noteworthy that the experimental conditions (concentrations of the treatments) can possibly influence the signal relationships depicted here. However, in previous work [20] we published the transcriptome response of treatments of C and N at different concentrations (NO_3_^−^ at 0, 5, 10 and 15 mM) and Carbon (Sucrose at 0, 30, 60 and 90 mM). In that analysis, we found no dose effect of the signals on gene expression. This supports our simplification of gene expression patterns as binary patterns.

### Signal integration overview in *Arabidopsis*

The role of autotrophic leaves as an energy converter has been known since the 18^th^ century. Shoots of plants capture solar energy and convert it into sugars through photosynthesis, thereby constituting the major entry of energy into food chains. Our current findings showed that the management of signal integration and their consequences on a genome-wide scale follow this centuries-old paradigm. Our study shows that signal integration, for the considered signals, is more important in roots than in leaves. In photosynthetic leaves, the main signal in the control of gene expression is L. We also show that the L signal in leaves is insensitive to C, N or combinations thereof (Figure 6B). Corresponding L-controlled genes in leaves have significantly over-represented functions including metabolism and photosynthesis (data not shown). By contrast, in the heterotrophic roots, L is very poorly sensed on its own (Figure 5A, C-e), and L and C act on genes in an unexpectedly highly coordinated fashion (Figure 7). Our genome-wide study also suggests that sensing systems in heterotrophic roots are very responsive to the presence of sugar, whether this resource comes from an externally supplied source or from leaves as photosynthate. Recent findings on root ion transporters support this hypothesis, by showing that 16 out of 19 light- or carbon-regulated transporters were directly controlled by a carbon signaling pathway [18]. Moreover, we showed that the CL composite signal exerts a negative feedback loop on the actions of C and L. This loop means that gene regulation by C or L reaches a plateau and the CL signal does not have any synergistic effect on gene expression control. This observation reinforces the notion that roots primarily sense L as C. More interestingly, we found a pronounced effect of CN as a repressor of C or L signals (Figure 7 and Figure 8 panels e–f). This repression corresponds to genes controlled by C or L, for which control is disrupted (the level of the CN coefficient is equal to the C effect) by the presence of CN. In other words, these 136 genes (Figure 7 and Figure 8, panels e and f) are under the control of a yet-to-be-identified C and N sensing system and are up- or down-regulated only when C but not N is applied to plants. This type of genomic regulation might correspond to the signaling evoked by Moore et al. (2003) for photosynthetic genes [12]. Indeed, sugar repression of *CAB1* and *RBCS* are antagonized by nitrate. These newly discovered candidate genes as a group will deserve further analysis to identify the molecular mechanisms involved in their control and consequently elements of the C and N sensing system.

In conclusion, this analysis provides mathematical models that explain global gene expression as a function of C, N and L in roots and leaves. Analyses of the models provided insights into nutrient signal transduction pathways in a sessile organism, *Arabidopsis*. Our findings provide a new model of C, N and L signal management and suggest that many of the effects seen for single genes [12],[18],[19],[36],[37],[38], are in fact managed by the plant at a systemic level (Figure 7, Figure 8). We believe that our findings have broad relevance since not only are plants the primary providers of C and N through sugar and amino acid biosynthesis, but also carbon fixation via photosynthesis is a major factor that can help alleviate global warming. In this context, understanding systematic C/N/L signal interaction at a genomic scale in plants may provide new ways to tackle agricultural productivity and other socio-economical and environmental problems.

## Materials and Methods

### Plant culture and transcriptome analysis

Arabidopsis thaliana Col-0 were grown hydroponically in nutrient solution as described previously [20]. To summarize, plants were directly grown on cut eppendorf tubes which had mesh at the bottom and were filled with sand. These tubes were placed in custom-designed styrofoam rafts floating on a nutrient solution, in a growth chamber (EGC, Chagrin Falls, OH, USA) at 22°C with 60 µmol.m^−2^.s^−1^ light intensity and 8 h/16 h light/dark cycles. The seeds were initially germinated in tap water for one week, then transferred to a complete nutrient solution, which was renewed weekly [7]. After six weeks, plants were transferred to fresh media the day before the experiments. For treatments, individual rafts were transferred to containers with 300 ml of nutrient solution supplemented with various concentrations of nitrate [as a mix of 2/1 KNO_3_/Ca(NO_3_)_2_] and/or sucrose. The N-free nutrient solutions contained 0.25 mM K_2_SO_4_ and 0.25 mM CaCl_2_ instead of KNO_3_/Ca(NO_3_)_2_. Plants were transferred to treatment media at the beginning of the light period and were harvested 8 h afterwards. Roots and leaves were collected separately and quickly frozen in liquid nitrogen.

### Microarray hybridization

Total RNA extraction was performed as described previously [20]. Briefly, cDNA were synthesized from 8 µg total RNA using T7- Oligo(dT) promoter primer and reagents recommended by Affymetrix (Santa Clara, CA, USA). Biotin-labeled cRNA was synthesized using the Enzo BioArray HighYield RNA Transcript Labeling Kit (Enzo, New York, NY). The concentration and quality of the cRNA were evaluated by A260/280 nm reading and 1% agarose gel electrophoresis. We used 15 µg of labeled cRNA to hybridize the *Arabidopsis* ATH1 Affymetrix gene chip for 16 h at 42°C. Washing, staining and scanning were performed as recommended by Affymetrix. Image analysis and normalization to a target median intensity of 150 was performed with the Affymetrix MAS v5.0 set at default values. We analyzed the reproducibility of replicates using the correlation coefficient and visual inspection of scatter plots of pairs of replicates. One pair of duplicates failed this quality control. Thus, to improve the reliability of the measure we performed two more Affymetrix chips from independent samples corresponding to the condition: roots, light, no nitrogen, and no carbon.

### Modelling of gene expression patterns

All data manipulations were performed in R (http://www.r-project.org/). The ANOVA analysis was carried out using the R *lm()* function with three models. The first model considers the organs as a factor, such that the expression Y_i_ of a gene_i_ is given by: Y_i_ = α_0_+α_1_C+α_2_L+α_3_N+α_4_O+α_5_CL+α_6_CN+α_7_CO+α_8_LN+α_9_NO+α_10_LO+α_11_CNL+α_12_LNO+α_13_CNO+α_14_CLO+α_15_CLNO+Z. In this model, α_0_ represents the expression under a “control” condition (without C, without N, without L, in roots); *Z* represents the noise; and α_1_ to α_15_ represent the coefficients quantifying the effect of each factor (C, N, L, O) or combination of factors. The second model is a simplified version of the first model in which gene expression in roots and leaves datasets were analyzed separately: Y_i_ = α_0_+α_1_C+α_2_L+α_3_N+α_4_CL+α_5_CN+α_6_LN+α_7_CNL+Z. Each gene was analyzed separately. We addressed multiple testing by controlling the false discovery rate (FDR) at 1% at each stage of the evaluation procedure as described previously [20]. A rigorous statistical procedure was implemented to avoid over-fitting. The complete models were used to assess whether gene expression could be explained at all by any combination of the coefficients. If the model was significant at 1% FDR, then each significant term in the model was evaluated to determine if its presence contributed to the final model. Terms with higher p-values were tested first. We used the anova() function to compare models at each iteration of the procedure. Significant coefficients were organized as presented in supplemental Tables S1, S2, S3.

### Clustering algorithm, Sungear analysis, and interpretations

Hierarchy between signals were evaluated by average linkage hierarchical clustering. First, euclidian distances were calculated using the *dist()* function in the R software. Second, clusters were generated by the *hclust()* function. Third, plots were generated using the *plot()* (default values) function. Dendrogram interpretations were carried out as previously described [26]. Concept: the fact that a given gene behave similarly in response to 2 factors (example: C and L), will increase the linkage of those 2 factors (decrease the distance). Hence, at a gene list (genome) scale, the study of dendrograms allows to visually capture the relative relationship of the signals in the control of the considered gene set regulation. Note that branch length is set to a constant value and is not related to the data (*plot()* function with default values). Only the height of the node reflects the distance between the branches and the associated leaves of the tree.

Because the dendrograms do not give any direct information on the size of the gene sets or their overlaps, we used Sungear software [28] as a complement. We sorted genes for which a given signal had a positive call. Then, the corresponding gene lists were uploaded via the VirtualPlant online interface (http://www.virtualplant.org). The Sungear software (can be understood as a generalized Venn Diagram) displays polygons with the signals at the vertices (anchors). The circles inside the polygon (vessels) represent the genes controlled by different signals as indicated by the arrows around the vessels. The area of each vessel (size) is proportional to the number of genes associated with that vessel. Thus, by visually analyzing the figure we can directly evaluate the signal interactions.

## Supporting Information

Figure S1 Hierarchical clustering of the magnitude of the model coefficients reveals relationships between signals. Average linkage hierarchical clustering with euclidean distance was used to analyze the model coefficient matrices for the entire data set (A, Table S1), leaves data set alone (B, Table S2), roots data set alone (C, Table S3).
(0.08 MB PDF)

Figure S2 Example of genes controlled in roots or in shoots by combination of factors. Genes found to be controlled by a combination of factors by our modeling approach (as the only signal, see Figure 1 for a definition) were sorted. The expression pattern of one representative gene belonging to each category is presented. Asterisks indicate conditions captured in the model of gene expression. Note that for At5g36950, the strong variability in the carbon treatment in light (first yellow bar) does not allow the analysis to detect C as a significant effect.
(0.13 MB PDF)

Table S1 (1.47 MB XLS)

Table S2 (0.35 MB XLS)

Table S3 (0.14 MB XLS)

Table S4 (0.15 MB XLS)
