# Supplementary material for: A Systems Approach Uncovers Restrictions for Signal Interactions Regulating Genome-wide Responses to Nutritional Cues in Arabidopsis
Source: PLoS Comput Biol. 2009 Mar 20;5(3):e1000326. doi: 10.1371/journal.pcbi.1000326 (PMC2652106; doi:10.1371/journal.pcbi.1000326)
Supplement: Figure S1 — Hierarchical clustering of the magnitude of the model coefficients reveals relationships between signals. Average linkage hierarchical clustering with euclidean distance was used to analyze the model coefficient matrices for the entire data set (A, Table S1), leaves data set alone (B, Table S2), roots data set alone (C, Table S3). (0.08 MB PDF) [file pcbi.1000326.s001.pdf]

Figure S1

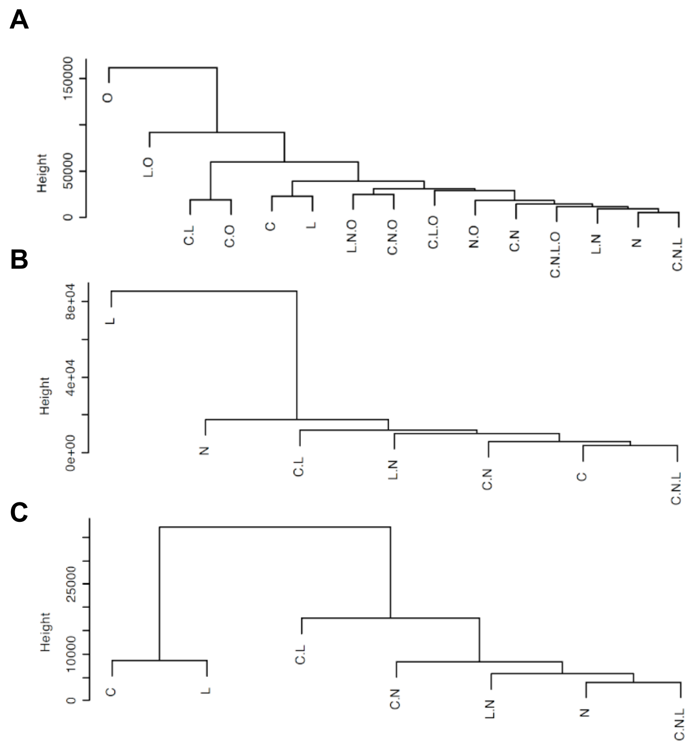

Figure S1 Hierarchical clustering of the magnitude of the model coefficients reveals relationships between signals.
Average linkage hierarchical clustering with euclidean distance was used to analyze the model coefficient matrices for the entire data set (A, Table S1), leaves data set alone (B, Table S2), roots data set alone (C, Table S3).
