# Supplementary material for: A Systems Approach Uncovers Restrictions for Signal Interactions Regulating Genome-wide Responses to Nutritional Cues in Arabidopsis
Source: PLoS Comput Biol. 2009 Mar 20;5(3):e1000326. doi: 10.1371/journal.pcbi.1000326 (PMC2652106; doi:10.1371/journal.pcbi.1000326)
Supplement: Figure S2 — Example of genes controlled in roots or in shoots by combination of factors. Genes found to be controlled by a combination of factors by our modeling approach (as the only signal, see Figure 1 for a definition) were sorted. The expression pattern of one representative gene belonging to each category is presented. Asterisks indicate conditions captured in the model of gene expression. Note that for At5g36950, the strong variability in the carbon treatment in light (first yellow bar) does not allow the analysis to detect C as a significant effect. (0.13 MB PDF) [file pcbi.1000326.s002.pdf]

Figure S2

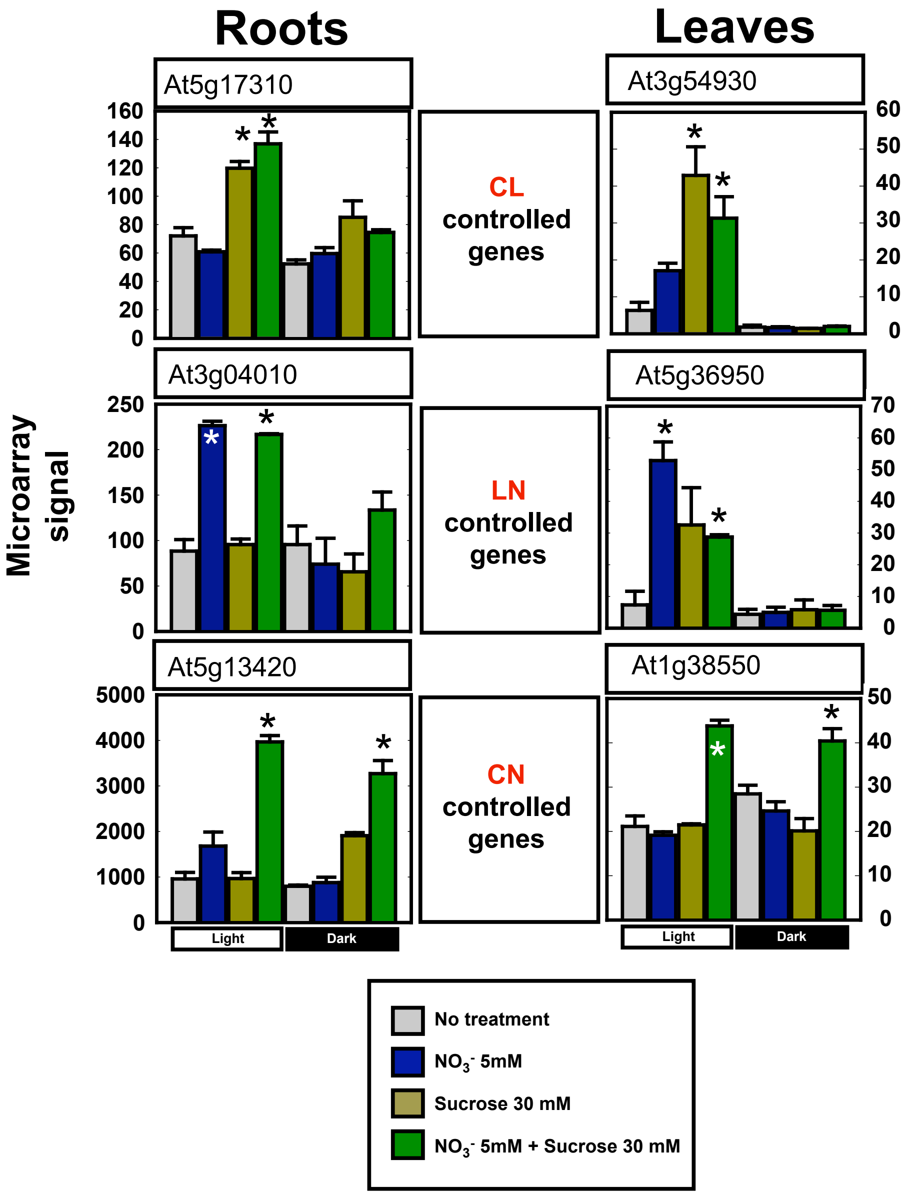

Figure S2 Example of genes controlled in roots or in shoots by combination of factors. Genes found to be controlled by a combination of factors by our modeling approach (as the only signal, see Figure 1 for a definition) were sorted. The expression pattern of one representative gene belonging to each category is presented. Asterisks indicate conditions captured in the model of gene expression. Note that for At5g36950, the strong variability in the carbon treatment in light (first yellow bar) does not allow the analysis to detect C as a significant effect.
